# Threshold-Filtered Kinetic Monte Carlo Simulation for Real-Time Simulation and Control of Biomass Fractionation

**DOI:** 10.1021/acs.iecr.5c04698

**Published:** 2026-03-13

**Authors:** Juhyeon Kim, Jiae Ryu, Qiang Yang, Chang Geun Yoo, Joseph Sang-Il Kwon

**Affiliations:** † Artie McFerrin Department of Chemical Engineering, 14736 Texas A&M University, College Station, Texas 77845, United States; ‡ Texas A&M Energy Institute, 14736 Texas A&M University, College Station, Texas 77845, United States; § William G. Lowrie Department of Chemical and Biomolecular Engineering, Columbus, Ohio 43210, United States; ∥ Department of Chemical Engineering, State University of New York College of Environmental Science and Forestry, Syracuse, New York 13210, United States; ⊥ School of Packaging, 3078 Michigan State University, East Lansing, Michigan 48824, United States

## Abstract

For lignin and other polymer reaction systems, reaction kinetics are inherently complex. Individual bond-cleavage reactions exhibit a wide distribution of activation energies due to structural heterogeneity among β-*O*-4 linkages. In conventional kinetic Monte Carlo (kMC) simulations, reactions with high activation energies, whose probabilities are extremely low, are still evaluated at every step, leading to substantial computational cost with negligible impact on system evolution. To overcome this limitation, we developed a threshold-filtered kMC framework that accelerates multiscale lignin fractionation simulations by excluding kinetically irrelevant events using an Arrhenius-type activation energy threshold. The model preserves its fidelity while reducing CPU time by orders of magnitude compared with conventional approaches. It accurately predicts the evolution of lignin molar masses and S/G ratios under varying reaction conditions. This strategy enables efficient, data-independent modeling of complex reaction networks and establishes a scalable tool for process-level optimization and control in lignin valorization systems.

## Graphical abstract

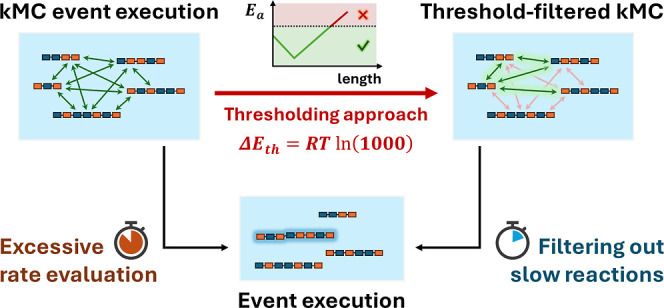

## 1 Introduction

The computational simulation of complex chemical processes presents a fundamental challenge in modern process engineering: achieving both high-fidelity system representation and computational efficiency simultaneously.^1,2^ This challenge becomes particularly critical in multiscale systems where phenomena occurring at vastly different temporal and spatial scales must be integrated within a unified modeling framework.^3–5^ Traditional simulation approaches often face computational bottlenecks that scale unfavorably with system size, creating significant barriers to real-time process control applications where rapid model evaluation and feedback are essential.^6^ For the polymer processes, moreover, the simulation domain continuously evolves due to chain growth, scission, and side reactions.^7–9^ Such an intricate reaction network that spans molecular to plant-scale dynamics intensifies the overall model complexity.^3,11^ As the complexity of system modeling increases to more accurately represent real-world processes, the computational cost also rises sharply, presenting a core challenge for real-time optimization and process control.^12–14^ Therefore, achieving both high prediction fidelity and operational efficiency requires carefully balanced modeling strategies to ensure that models remain both robust and computationally tractable.

Kinetic Monte Carlo (kMC) simulations have emerged as powerful tools for modeling stochastic chemical processes, specifically in polymer systems where discrete molecular events drive multiscale behavior.^15–18^ The strength of kMC lies in its ability to capture the probabilistic nature of molecular reactions while providing detailed insights into system evolution.^19–21^ A prominent example of such complexity is found in lignocellulose biomass processing,^22,23^ particularly during fractionation, where delignification, depolymerization, condensation, and demethoxylation all occur in the reactor. The biomass fractionation process involves multiscale events, including delignification and depolymerization.^24–26^ In this case, the layered kMC can also simulate such hierarchical reactions across multiple time and length scales, offering a comprehensive understanding of the overall process from interphase mass transfer to monomer-level chemistry.^27^ However, traditional kMC algorithms suffer from a critical computational limitation^28–30^ that becomes particularly severe in lignin systems. In particular, the number of dissolved chains sharply increases as depolymerization progresses. This changes the simulation domain throughout the operation, creating additional computational overhead. A growing population leads to a superlinear increase in the number of event candidates, rendering the simulation impractical for real-time control applications where rapid execution is essential for process optimization. Such challenges inherent to polymer kMC simulations have been recognized in related studies. For instance, convergence demands with respect to Monte Carlo control volumn have been systematically analyzed for PMMA depolymerization,^31^ highlighting the need for large simulation volumes to ensure numerical reliability. More recently, moment-driven kMC frameworks have been introduced to drastically reduce computational cost by operating on statistical moments,^32^ thereby facilitating faster integration with control-oriented modeling.

Computational acceleration of complex simulations has been pursued through multiple strategies, each addressing different aspects of the computational bottleneck problem.^33^ A scaling acceleration algorithm has successfully demonstrated its efficiency through continuum-kMC coupling strategies in radical polymerization systems.^34^ While it covered several case studies, including homo/copolymerization, it is currently limited to simple kinetic schemes and cannot handle intricate rate dependencies on evolving chain properties. When it comes to machine learning, reactive molecular dynamics approaches have been accelerated using neural network potentials, achieving significant speedup compared to ab initio calculations while maintaining chemical accuracy for thermal degradation.^35^ However, it remains computationally intensive for large-scale systems and requires extensive training data sets for different polymer compositions. Machine learning-enhanced kMC frameworks have shown substantial promise,^36–38^ with artificial neural network (ANN) accelerated approaches developed specifically for lignin fractionation achieving over 99% reduction in simulation time while enabling real-time model predictive control,^39^ yet this method is limited by its dependence on precomputed training data. Most critically for lignin fractionation systems, many existing acceleration strategies have been insufficient to address the fundamental algorithmic bottleneck: superlinear scaling of computational complexity with the number of reactive species. Once polymer scission is a dominant event, the number of macromolecular species increases exponentially as the reaction proceeds, and traditional kMC algorithms require evaluation of reaction rates for all possible combinations at each simulation step. This core limitation necessitates a different approach: one that achieves acceleration through algorithmic optimization rather than physical approximation or system-specific parametrization, while preserving the essential stochastic nature of lignin chemistry required for accurate property prediction.

In this study, we present a threshold-filtered kMC acceleration strategy tailored for the multiscale simulation of lignin fractionation. First, we consolidate all chain-level properties into an integrated data structure that updates only the chains impacted by an event, eliminating redundant rate evaluations and keeping instantaneous access to the reaction kinetics. Second, we utilize an Arrhenius-guided activation energy threshold to prune kinetically irrelevant events. Reactions that are significantly slower than the most favorable pathway are excluded from the event candidate, so the algorithm focuses on where it can actually change the system behavior. Third, we apply this rule consistently across microscopic reactions. Depolymerization rates for each chain are evaluated at the most labile sites and only candidates within the threshold. For condensation, the activation energy becomes a function of the combined molar mass of two condensing chains. Using the activation energy threshold, only chain-pair combinations whose combined length falls within the kinetically feasible window are considered. Together, these choices preserve stochastic fidelity while cutting rate evaluations by orders of magnitude, enabling high-resolution multiscale simulation and practical simulation acceleration.

The rest of this article is structured as follows: Section 2 showcases the kMC model configuration. Especially, from Section 2.3, the threshold-filtering-based model acceleration strategy is described in detail. Section 3 covers the controller design and closed-loop operation results. Afterward, we highlight the significance of the developed acceleration strategy with the concluding remarks in Section 4.

## 2 Model Formulation and Open-Loop Simulation

### 2.1 System Initialization

Lignin valorization relies on the effective fractionation of biomass, as the structure and chemical functionality of the resulting lignins govern their utility in downstream applications. One-stage organic solvent fractionation has emerged as a promising, cost-effective approach to reducing the heterogeneity of the properties of the resulting lignin products,^40–43^ facilitating their downstream applications.^44,45^ However, the quantitative relationship between fractionation conditions (e.g., solvent type, severity, or temperature) and the physicochemical properties most relevant for lignin conversion remains unclear, limiting rational process design. Further, these phenomena, ranging from delignification to depolymerization, take place simultaneously at multiple time and length scales. This inherent complexity has made it difficult for existing models to accurately predict how process conditions ultimately impact lignin structure and properties. As a multiscale challenge, this motivates the present work, which seeks to connect processing parameters, molecular evolution, and lignin valorization outcomes through a dedicated modeling framework.

Initially, the lignin chains only exist in the chip phase, and the algorithm initializes the system. The detailed initialization procedure is described in the Supporting Information. In this research, lignin is modeled as a linear macromolecule with two monolignols, S (syringyl) and G (guaiacyl) units, as our experiments were conducted using Aspen wood, which is a hardwood species. The average molar mass and S/G ratio of the pristine lignin chains are measured at 13,000 g/mol and 1.76, respectively. Based on this information, monolignol sequences are randomly assigned for each chain in the system. For model development, two temperature points were tested, which are 353 and 363 K. The experimental data of the pristine lignin and the fractionated lignin were adopted from the previous study.^27^ In brief, lignin fractionation was performed using 4-phenolsulfonic acid (72%) with Aspen wood chips (1 mm) at a 1:10 w/w solid-to-liquid ratio and at 353 and 363 K for 10–30 min. The cellulolytic enzyme lignin (CEL) was prepared according to the previous study^46^ and used as a native lignin in this study. The molar mass of lignin was measured by gel permeation chromatography (GPC) after dissolving acetylated lignin in tetrahydrofuran (THF).^27^ The detailed experimental setup and procedure can be found in our previous publication in detail.^27^

***Remark 1***. While native lignin is known to exhibit considerable branching and structural complexity,^47–50^ linear chain models have proven sufficient for predicting averaged properties such as molar mass distribution (or molecular weight distribution, MWd) and S/G ratios under process-scale conditions.^51–53^ In modeling applications, both strategies have been shown to be scientifically reliable, with the choice of model determined by the research goals and the specific characteristics.^54,55^ Beyond its scientific justification, adopting a simplified linear structure also offers clear practical benefits: it substantially lowers the computational demand while maintaining accuracy.^56^ Notably, numerous studies have demonstrated that linear chain models can provide reliable results, despite the known complexity of native lignin networks.

### 2.2 The Traditional kMC Simulation

Once the woody biomass (chip phase) is soaked in the reacting solvent (liquor phase), the biomass components dissolve out of the solid structure, becoming the liquor-phase elements. In this work, note that the term ‘liquor phase’ refers exclusively to the well-mixed liquid phase containing dissolved lignin species, for which all molecular properties are defined and analyzed throughout this paper. Specifically, lignin chains can be transferred in both directions, referred to as delignification and redeposition hereafter. These are classified as macroscopic processes and governed by the global mass balance equations, as shown below.

1 $$\begin{array}{ll} r_{D} & =-\frac{dL_{c}}{dt}=k_{D}L_{c}-k_{R}L_{d}\\ r_{R} & =-\frac{dL_{d}}{dt}=-k_{D}L_{c}+k_{R}L_{d} \end{array}$$

where *L*_c_ and *L*_d_ represent the mass of lignin in the chip and the dissolved lignin, and the subscripts *D* and *R* are for delignification and redeposition. Using the Arrhenius equation, the rate coefficients *k* for both processes can be expressed as follows

2 $$\begin{array}{ll} k_{D} & =A_{D}\;\exp\left(-\frac{E_{D}}{RT_{c}}\right)\\ k_{R} & =A_{R}\;\exp\left(-\frac{E_{R}}{RT_{f}}\right) \end{array}$$

where *A*_D_, *A*_R_, *E*_D_, and *E*_R_ denote the pre-exponential factors and activation energies for delignification and redeposition, respectively. According to the energy balance equations, the system temperature *T* changes, and the subscripts c and f stand for the chip and liquor phases. In the simulation, the dissolved mass of lignin increases over time. Once the cumulative dissolved mass of lignin surpasses the mass of one chain in the chip phase, the corresponding entry in the chip array is moved to the liquor array. Oppositely, redeposition also happens according to eq 1. Diffusional limitations are not explicitly resolved in the present framework. Instead, their effects are implicitly embedded in the effective rate coefficients, and the system is assumed to be well-mixed.

The temperature changes can be calculated using the following equations

3 $$\begin{array}{ll} \frac{dT_{c}}{dt}C_{P_{c}}M_{c} & =\Delta H_{R}r_{D}+U\left(T_{f}-T_{c}\right)\\ \frac{dT_{f}}{dt}C_{P_{f}}M_{f} & =-U\left(T_{f}-T_{c}\right)+C_{P_{ext}}{Ṁ}_{ext}\left(T_{ext}-T_{f}\right) \end{array}$$

where the subscript ext is for the external heat source, which is applied to maintain a constant liquor-phase temperature and used to control the system temperature to the desired range. *C*_P_ is the temperature-dependent heat capacity,^57^ *M* is the total mass of each phase, Δ*H*_R_ is the heat of the reaction, and *U* is the overall heat transfer coefficient. For the macroscopic processes, the time step is Δ*t* = 5 × 10^–4^ min. In each Δ*t*, lignin is dissolved out according to eqs 1–3.

The dissolved lignin chains can undergo three classes of reactions, including depolymerization, condensation, and demethoxylation. Unlike the macroscopic phenomena, these three happen between the adjacent chains. Therefore, these reactions occur on shorter time and length scales and are classified as microscopic reactions. They are much faster than the macroscopic ones, and consequently, multiple reactions are chosen and executed in the macroscopic time frame, Δ*t*. At the early stage, there can be some interactions between solid biomass and the solvent. However, this model is intended to explain only the transformation of dissolved lignin in the liquor phase. The initial solid-phase interaction was not considered in this work. In our framework, the microscopic time step (stochastic time increment) is calculated following the Gillespie direct method^58^ where δ*t* = −ln ξ/*r*_tot_ and ξ is a uniform random number drawn from the interval (0, 1). Note that *r*_tot_ represents the sum of all microscopic rates at a given moment (eq 7).

Depolymerization is a random chain scission reaction based on the activation energies along the chain. For *m*-th β-*O*-4 bond in chain *i*, the depolymerization rate becomes

4 $$r_{dep,im}=k_{dep,im}C_{L}\left(N_{i}\right)=A_{dep}\;\exp\left(-\frac{E_{dep,im}}{RT_{f}}\right)C_{L}\left(N_{i}\right)$$

where *C*_L_(*N*_*i*_) is the concentration of the dissolved chain *i*. To calculate the depolymerization rate, the activation energy value is necessary, and a particular activation energy value is assigned to each β-*O*-4 bond^27^ based on its S/G sequence. *E*_dep,im_ values are stored in the library in terms of temperature and monolignol sequence.

Condensation is a merging event where two chains are involved, and the rate is calculated as follows

5 $$r_{con,ij}=k_{con,ij}C_{L}\left(N_{i}\right)C_{L}\left(N_{j}\right)=A_{con}\;\exp\left(-\frac{E_{con,ij}}{RT_{f}}\right)C_{L}\left(N_{i}\right)C_{L}\left(N_{j}\right)$$

According to the DFT calculation, *E*_con,*ij*_ is influenced by the combined mass of chains *i* and *j*. To evaluate the condensation rates for all combinations, the molar masses of each combination need to be calculated. Condensation is modeled as a second-order reaction, as two chains participate in the merging event.

Demethoxylation is the transformation of one S unit to a G unit by removing one methoxy (−OCH_3_) group on the aromatic backbone, and its rate depends on the S content as shown below

6 $$r_{dem,i}=k_{dem}f_{S}C_{L}\left(N_{i}\right)=A_{dem}\;\exp\left(-\frac{E_{dem}}{RT_{f}}\right)f_{S_{i}}C_{L}\left(N_{i}\right)$$

where *f*_*S*_*i*__ is the *S* fraction in chain *i*. Since demethoxylation is the removal of one methoxy group that can happen in any chain, *E*_dem_ is considered as a constant value throughout the simulation, unlike *E*_dep,*i*_ and *E*_con,*ij*_. Demethoxylation is generally difficult to achieve without severe conditions, such as using catalytic hydrogenation.^59^ However, the change in S/G ratio is hard to explain without demethoxylation, which is the transformation of one S unit to a G unit by removing one methoxy (−OCH_3_) group on the aromatic backbone. We hypothesize that this reaction can occur in this acidic fractionation, and its rate depends on the *S* content.

From a kinetic perspective, the combined effect of chain length and steric effects could be interpreted as entropic contributions to the activation energies. In this work, these effects are incorporated into an effective activation energy. This choice is motivated by the fact that the DFT-derived barrier corresponds to the transition-state geometry, in which restricted configurational freedom and steric constraints are already implicitly embedded. The numerical values of the activation energies used in this work are adopted from our previous high-fidelity kMC study,^27^ where their physical basis and calculation procedures are discussed in detail. The present work focuses on their algorithmic utilization and multiscale integration. Note that we set the pre-exponential factors as constants: *A*_dep_ = 4.8 × 10^22^, *A*_con_ = 2.5 × 10^20^, and *A*_dem_ = 9.5 × 10^104^ min^–1^. Demethoxylation is regarded as a simple phenomenon that takes one methoxy group in a given chain, so a constant value of *E*_dem_ = 764 kJ/mol is used, considering its substantially high sensitivity against the reaction temperature. While *E*_dep_ values are used as a discrete value for each lignin chain of different S/G configurations, *E*_con_ is expressed as a function of the reaction temperature and the molar mass of the combined lignin chain (refer to Section S2 in the Supporting Information).

To realistically simulate the system over time, one needs to execute the most probable events and represent the accurate distributions in the resulting lignin properties. In this case, for the dissolved lignin chains, one of the microscopic changes occurs for an arbitrary chain in the system. The kMC algorithm evaluates the event selection probabilities based on the reaction rate distribution from the current system configuration. In the above traditional kMC algorithm, it evaluates the reaction rates for the chains in the liquor phase and then forms the overall rate distribution

7 $$r_{tot}=\sum_{i}r_{dep,i}+\sum_{i}\;\sum_{j\neq i}r_{con,ij}+\sum_{i}r_{dem,i}$$

Subsequently, a random number is generated for a probabilistic event execution, with a trend that the faster reaction has the higher chance of execution.

A schematic diagram of the simulation is present in Figure 1.^27^

**Figure 1:**
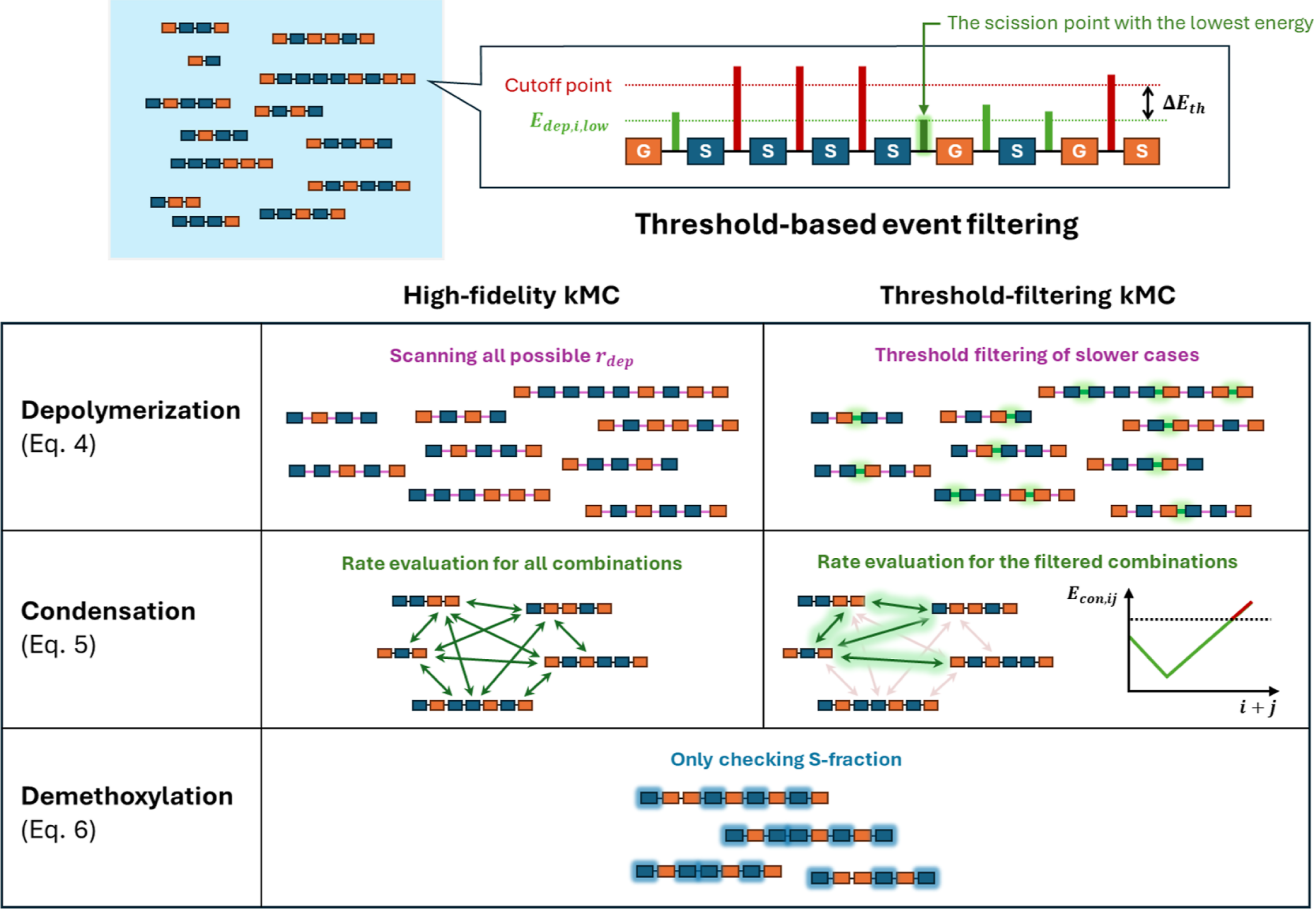
Schematic comparison of the traditional kMC algorithm (left) and the developed threshold-filtering kMC (right). While the traditional approach evaluates reaction rates for all possible events at each step, the threshold-filtering method applies activation energy criteria (eq 9) to exclude kinetically irrelevant events before rate evaluation.

### 2.3 Threshold-Filtered kMC Acceleration Strategy

However, when it comes to the high-fidelity kMC simulation, every microscopic reaction event (depolymerization, condensation, and demethoxylation) requires explicit rate evaluation based on individual chain structures or chain-pair interactions. As the delignification progresses, new chains continue dissolving from the solid chip into the liquor phase. Moreover, the dissolved chains depolymerize to monomers or oligomers, thereby increasing the total population of reactive species over time. Consequently, the number of rate evaluations grows, leading to a steep rise in computational cost at every time step.

To overcome this limitation and make the simulation framework computationally tractable, a threshold-filtered acceleration strategy is introduced. This method streamlines microscopic event generation by discarding kinetically insignificant events through activation energy-based filtering, while still preserving accurate system-level dynamics. As a result, the model retains the detailed chemical specificity of the high-fidelity kMC but achieves a substantial reduction in computational time, enabling computationally efficient dynamic simulations of lignin fractionation systems with minimal accuracy loss. The performance of the threshold-filtered kMC framework is quantitatively benchmarked against the existing kMC approaches in Section 2.4, covering both computational cost and all key molecular properties, including the average molar masses (*M*_n_ and *M*_w_), and the S/G ratio. In addition, a schematic illustration of the overall simulation process with the detailed explanation can be found in the Supporting Information.

#### 2.3.1 Data Integration

Figure 1 illustrates the workflow comparison between the conventional kMC and the developed threshold-filtering approach. In the conventional kMC approach (left panel), the algorithm needs to access all the dissolved chains to retrieve kinetic parameters. To construct *r*_tot_ at each step, one needs to evaluate each chain (for depolymerization and demethoxylation) and each chain pair (for condensation), accounting for a significant computational demand.

Conversely, in our framework, all important properties are continuously tracked and stored within the data structure, rather than scanning chains every time. To achieve this, at the initialization step, diverse properties for each chain are stored in the data structure, including S/G sequence (for S/G ratio calculation), associated *E*_dep,im_ for β-*O*-4 cleavage (eq 4), chain length (for rate calculation, eqs 4–6), MW (for eq 5 and MWd calculation), mass (for macroscopic calculations, eq 1), and S-unit fraction (for eq 6). This effectively bypasses the need for chain evaluation at each time step. A graphical illustration and its working principle are described in Section S3 in the Supporting Information. Once a microscopic reaction happens, one only needs to update the selected chains with all the above properties, efficiently handling the whole system evolution without unnecessarily repeating iterative calculations.

#### 2.3.2 Threshold Filtering

The proposed algorithm operates in two conceptually distinct steps: event filtering and stochastic sampling. Importantly, stochastic event execution is always performed based on reaction rates, exactly as in the standard kMC formulation. The activation energy criterion, which is described below, is introduced solely in the first step, where it is used to prefilter kinetically irrelevant events prior to any rate evaluation. Note that reaction rates depend not only on intrinsic kinetics but also on dynamically evolving concentrations and chain populations, which would require full rate evaluation at every step and thus defeat the purpose of acceleration. In contrast, activation energies are event-intrinsic quantities that vary slowly or remain fixed over the lifetime of an event. Owing to the Arrhenius relationship, a sufficiently large difference in activation energy guarantees an exponential separation of time scales, allowing events with negligible contributions to the total rate to be excluded a priori.

The Arrhenius expression, *k* = *A* exp­(−*E*/*RT*), describes the relationship between the rate coefficient and activation energy. In this work, for each reaction type, the events whose rate coefficients are 1000 times smaller than the fastest case are considered to contribute negligibly to *r*_tot_ calculation. If one compares the faster and slower reactions, we have

8 $$\frac{k_{fast}}{k_{slow}}=\exp\left(\frac{-E_{slow}+E_{fast}}{RT_{f}}\right)$$

Assuming *k*_slow_ = *k*_fast_/1000, eq 8 becomes

9 $$\Delta E_{th}=E_{fast}-E_{slow}=RT_{f}\;\ln1000$$

At 353 K, the calculated threshold is 20.28 kJ/mol, and as the system temperature changes (eq 3), the corresponding Δ*E*_th_ is adjusted using eq 9. In the high-fidelity simulation, depolymerization and condensation trigger a large number of rate calculations. For both reactions, the developed approach efficiently filters out the kinetically irrelevant events and significantly reduces CPU time throughout the simulation. It should be noted that the stochastic event selection procedure itself remains unchanged. Event probabilities are sampled proportionally to their reaction rates, as in the conventional kMC algorithm. The threshold-filtering strategy only reduces the candidate event set prior to rate evaluation, without altering the underlying probability sampling mechanism.

After this energy-based filtering, the reaction rates are evaluated for the retained events and normalized to form the standard rate-proportional selection probabilities. Subsequently, unbiased stochastic sampling is carried out using the standard rate-proportional selection rule. As a result, the activation energy threshold provides a provable upper bound on the contribution of excluded events while preserving exact rate-based sampling among the kinetically relevant events.

***Remark 2***. While the Arrhenius rate constant depends on both the activation energy and the pre-exponential factor, the proposed work focuses on resolving activation energies through AIMD and DFT-based analysis, as they dominate the exponential sensitivity of reaction rates. Pre-exponential factors are treated as reaction-specific constants, consistent with common practice in lignin and polymer kinetics,^60–63^ where their systematic dependence on macromolecular structure is difficult to resolve. This modeling choice enables reduction of kinetic degrees of freedom while retaining physically meaningful rate hierarchies and computational tractability.

#### 2.3.3 Depolymerization

In the liquor phase, each dissolved chain possesses a unique sequence of S- and G-type monolignols, which directly influences *E*_dep_ associated with β-*O*-4 bond cleavage. For chain *i*, the algorithm first identifies the lowest activation energy among its scission sites, which is denoted as *E*_dep,*i*,low_ (refer to the top of Figure 1). For the dissolved chain *i*, *E*_dep,*i*_ is continuously being tracked by the structured data table throughout the simulation. In this work, only the scission site candidates satisfying the following threshold condition are considered relevant for the event selection process

10 $$E_{dep,im}\leq E_{dep,i,low}+\Delta E_{th}$$

According to the above, bonds whose activation energy exceeds this threshold are excluded from rate evaluation, as their contribution to *r*_tot_ is kinetically negligible under the given reaction temperature. This filtering significantly reduces the number of evaluated bond-scission events per chain. Thus, it allows the model to capture dominant β-*O*-4 cleavage pathways with minimal computational overhead.

#### 2.3.4 Condensation

Condensation occurs when two lignin chains link together to form larger structures, counteracting the depolymerization process. From our previous work,^27^ various model structures were used to calculate the activation energy (*E*_con,*ij*_). In the simulation framework, *E*_con,*ij*_ between chains *i* and *j* is determined as a function of the combined molar mass (MW_*i*+*j*_), which is obtained from regression of first-principle (DFT) calculations. *E*_con,*ij*_ reaches its minimum when MW_*i*+*j*_ = 972 g/mol and increases linearly in both smaller and larger MW ranges. Since the molar masses of S and G monolignols are 227.2 and 179.2 g/mol, *E*_con,*ij*_ has the lowest value at the combined chain length of 4–5. An upper bound of the combined MW is determined based on the activation energy threshold as shown in eq 11.

11 $$E_{con,ij}\leq E_{con,ij,low}+\Delta E_{th}$$

At 353 K, eq 11 is satisfied for the MW range of MW_*i*+*j*_ ≤ 1672.33. If one assumes that the condensed chain only consists of the G units, it would have the maximum chain length of 9.3. Thus, for condensation, cases that make 2 ≤ *i* + *j* ≤ 9 are considered for rate calculation. It must be noted that *E*_con,*ij*_ is defined as a function of the combined chain length in this work, regardless of the structural differences. Consequently, the computational load decreases from evaluating all pairwise combinations to a very limited set of physically meaningful interactions, while preserving the statistical structure and mechanistic accuracy of condensation kinetics.

### 2.4 Model Performance

The degree of delignification is a direct indicator of biomass fractionation and also impacts the depolymerization kinetics. The molar masses of the dissolved chains primarily influence the physical properties determining their downstream applications. Moreover, different S/G sequences lead to distinct lignin reactivities. Consequently, tracking the above three variables is of paramount importance to optimize and regulate lignin properties in practice.

The delignification is influenced by the reaction temperature, reaction time, and chip size.^25^ As shown in Figure 2, the lignin contents decreased with the reaction time from 10 to 30 min at 353 and 363 K. Note that, due to the potential redeposition of the dissolved lignin, the lignin content converges to specific points. The macroscopic behavior above also affects the microscopic properties of lignin.

**Figure 2:**
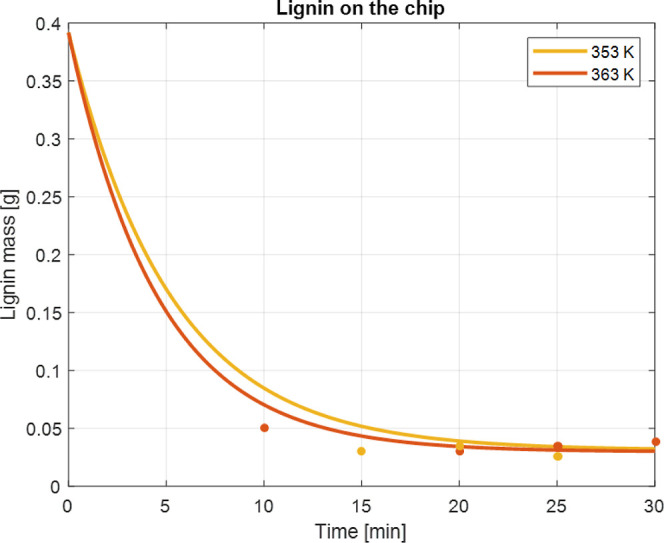
Remaining lignin mass on the chip phase at 353 and 363 K.

Figure 3 shows the key lignin properties, including the molar masses and S/G ratios. Among the microscopic reactions, depolymerization primarily affects the lignin populations. This results in a decreasing trend in average molar masses at both temperatures. Under acidic conditions, the ether linkage of lignin can be cleaved by the acid, promoting the isolation of lignin along with the depolymerization.^64^ On the other hand, condensation reactions of the depolymerized lignin can occur via electrophilic substitution, forming C–C bonds.^65^ In the liquor phase, the population of short chains increases rapidly due to fast depolymerization, and this influences the delignification and redeposition kinetics. According to Figure 2, delignification proceeds more rapidly at a higher temperature, resulting in more long chains being released from the bulk biomass into the liquor phase during the first 15 min. Consequently, a larger fraction of longer chains is present at the early stage. As shown in Figure 3, the average molar mass tends to be higher in the 363 K case during the first 5 min. However, since depolymerization is also accelerated at elevated temperatures, the average molar mass gradually decreases as delignification slows down. It can also be explained by a larger difference in *M*_n_ and *M*_w_. When it comes to the S/G ratio, the developed accelerated model successfully captured the high temperature sensitivity as well. Overall, our thresholding approach works well in estimating lignin properties throughout the reaction.

**Figure 3:**
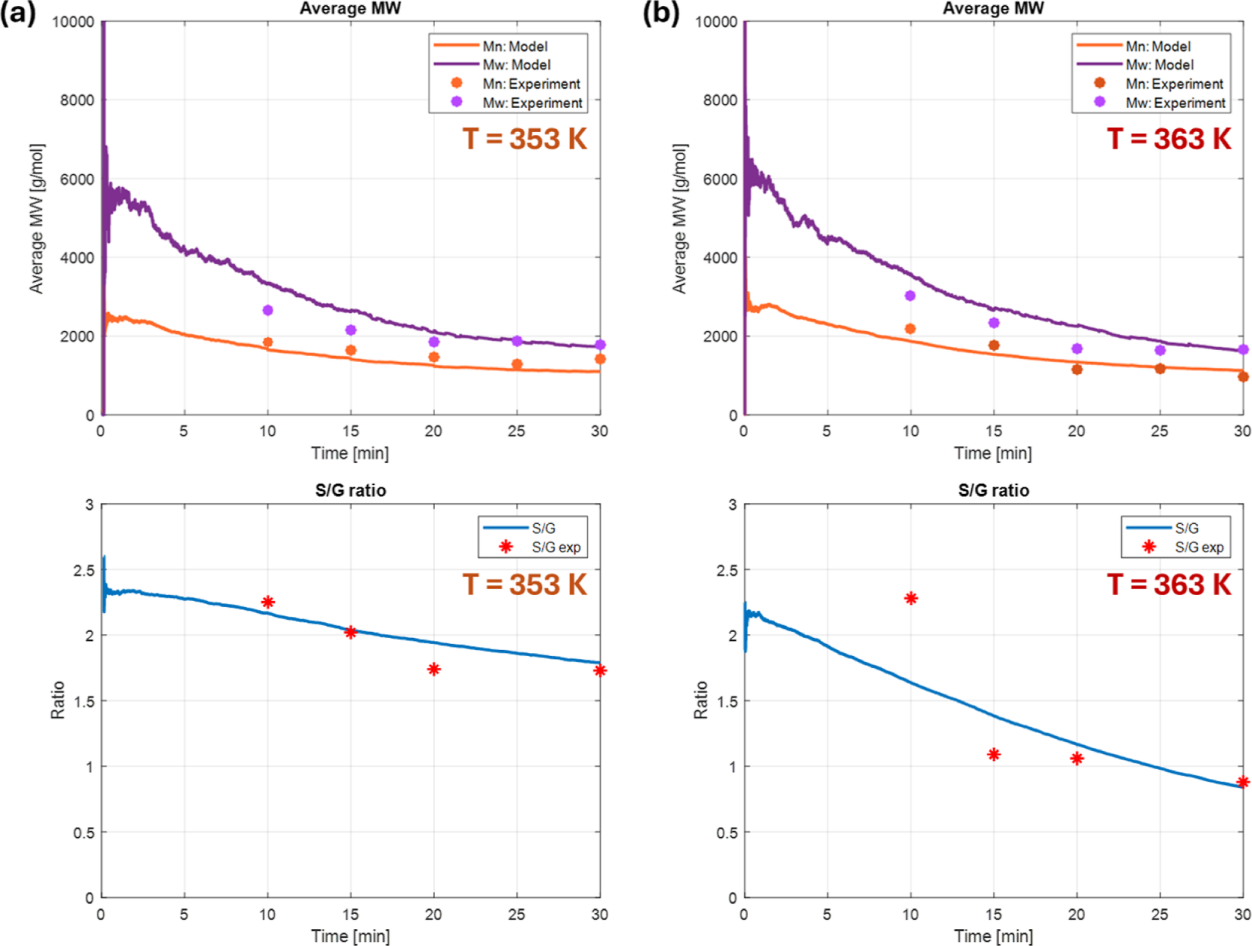
(Top) The average molar masses and (bottom) the S/G ratio tracked by the model under (a) 353 and (b) 363 K, respectively.

In Figure 4, simulation performance and model accuracy among three computational frameworks are comprehensively compared: the original high-fidelity kMC,^27^ the ANN-accelerated kMC,^39^ and the accelerated kMC model developed in this study, under the identical environment. As depicted in Figure 4a, our approach drastically reduces CPU runtime compared to the other two methods, highlighting its suitability for real-time control and large-scale simulations, which are essential for further applications. The original kMC model, while providing rigorous stochastic fidelity, exhibits prohibitively high computational cost, limiting its applicability to real-time process control. The ANN-accelerated kMC significantly decreases simulation time by utilizing pretrained neural networks to supplement reaction event selection, but its effectiveness can be restricted by dependence on system-specific training data and limited generalizability to new reaction conditions. In contrast, the developed framework achieves a substantial reduction in CPU runtime relative to the classical kMC approach, while simultaneously preserving algorithmic flexibility and eliminating reliance on precomputed data sets. This improvement is attributed to key algorithmic strategies, including population-based chain sampling, integrated data structures that avoid redundant scanning, and targeted prioritization of kinetically relevant reaction sites. These features collectively enable rapid and scalable stochastic simulations for detailed molecular property tracking.

**Figure 4:**
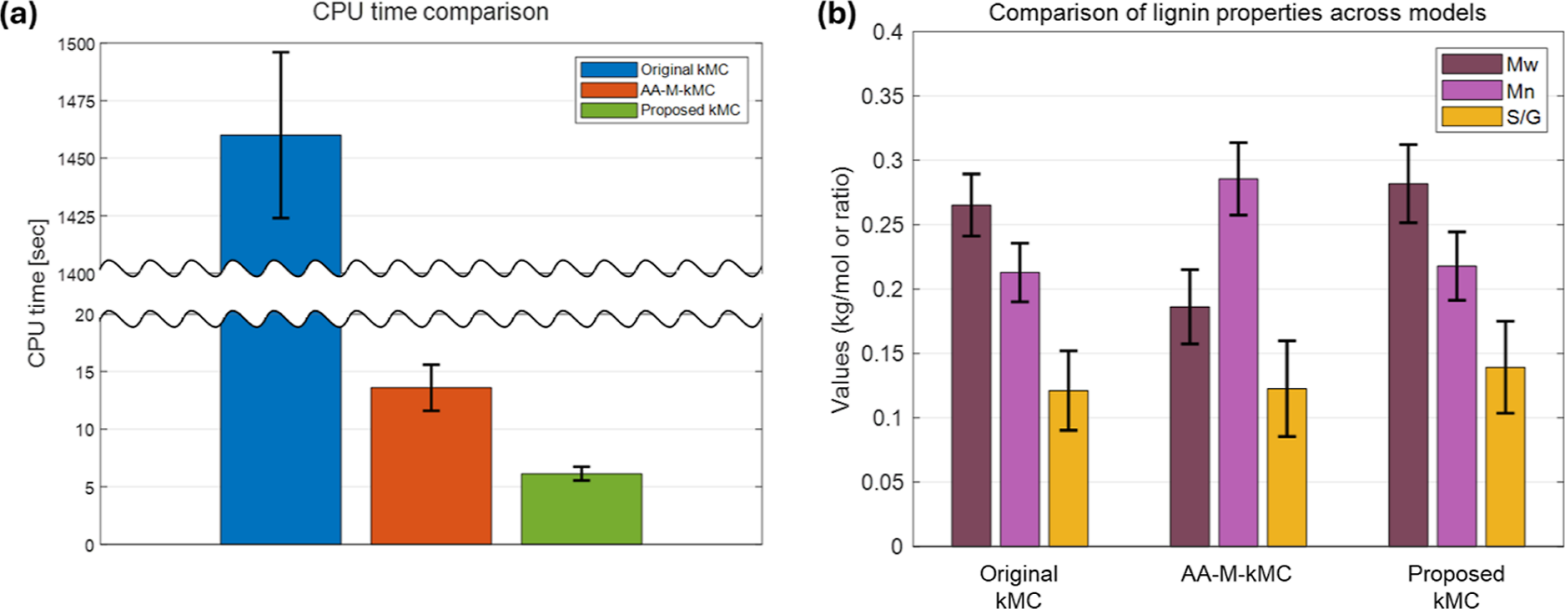
(a) CPU time comparison among three kMC frameworks: original high-fidelity kMC (blue^27^), ANN-accelerated kMC (orange^39^), and the developed framework (green). (b) Relative prediction errors of key lignin properties (molar mass and S/G ratio) from each model.

The quantitative error comparison in Figure 4b across all three models for *M*_n_, *M*_w_, and the S/G ratio confirms that the accelerated kMC model maintains high accuracy in tracking key lignin properties relative to experimental data sets and compared to the other methods. For all three models, the error ranges for the average molar masses remain at the one-monomer level, around 250–300 g/mol, and the error in the S/G ratio can be considered minimal. Notably, the developed strategy shows consistently low errors, closely matching the results of the other two. This robustness establishes not only the efficiency but also the reliability of the developed framework for predicting lignin properties, addressing bottlenecks in multiscale simulation of the fractionation process. The model’s ability to deliver reliable molecular property predictions with minimal computational overhead underlines its potential for integration into process optimization and model predictive control systems in biorefinery contexts. While the proposed model exhibits its predictive capability, the additional reactions in the reacting solvent, such as solid–liquid interaction in the early-stage fragment, and dissolution performance of condensed lignin, can be considered to further tune the model.

## 3 Model Predictive Controller (MPC) Design

### 3.1 Control Formulation

The microscopic reactions significantly alter the key properties of the resulting lignin chains. Their reaction kinetics are primarily affected by the reactor temperature. Hence, to optimize the lignin properties as desired, the reactor temperature can be controlled. In this study, according to eq 3, the external heat jacket is utilized to adjust the reactor temperature. In the MPC, temperature control will be directly performed by *T*_ext_, and the slope will be handled by ${Ṁ}_{ext}$ . Consequently, *M*_w_ and the S/G ratio will be regulated.

Regarding the above, the MPC is formulated as shown below

12 $$\begin{array}{l} \min_{T_{ext,k}}\sum_{p=1}^{2}\omega_{p}{\left(X_{p}\left(t_{N}\right)-X_{p,sp}\right)}^{2}\\ s.t.\;X_{p}=f_{p}\left(T_{ext},{Ṁ}_{ext}\right)\left(Accelerated\;kMC\;model,Section2\right)\;\\ \begin{array}{l} 343\leq T_{ext}\leq 363\\ 40\leq {Ṁ}_{ext}\leq 100\\ \left|T_{ext}\left(t_{k+1}\right)-T_{ext}\left(t_{k}\right)\right|\leq 5\forall k\in \left\{1,...,N-1\right\} \end{array} \end{array}$$

where ω_*p*_ is a weight constant for each controlled output **X**_*p*_ (*M*_w_ and the S/G ratio). The process measurements are provided every 5 min, so the number of the prediction horizon (*N*) becomes 6. The accelerated model above is integrated to predict the desired *T*_ext_ and ${Ṁ}_{ext}$ profile in a real-time fashion based on the process measurements.

### 3.2 The Closed-Loop Control Results

Using the developed controller, important lignin properties are controlled. The virtual experiment is conducted with the high-fidelity kMC model. The key observations from the experiments are that the depolymerization reaction is dominant over the other microscopic reactions, and the S/G ratio is highly sensitive to the temperature change.

The control inputs and outputs are shown in Figures 5 and 6.In this study, the set-points are set as *M*_w_ = 1500 g/mol, and S/G ratio = 1.50. To achieve this, the jacket temperature has been increased for the first 10 min, as indicated in Figure 5a. Moreover, as highlighted in Figure 5b, to heat the system rapidly, a jacket flow rate is set at the maximum value of 100 mL/min. At a high temperature, a number of long chains dissolve quickly within the first few minutes. At the same time, depolymerization rapidly breaks the chain. As a result, *M*_w_ and the S/G ratio substantially decrease.

**Figure 5:**
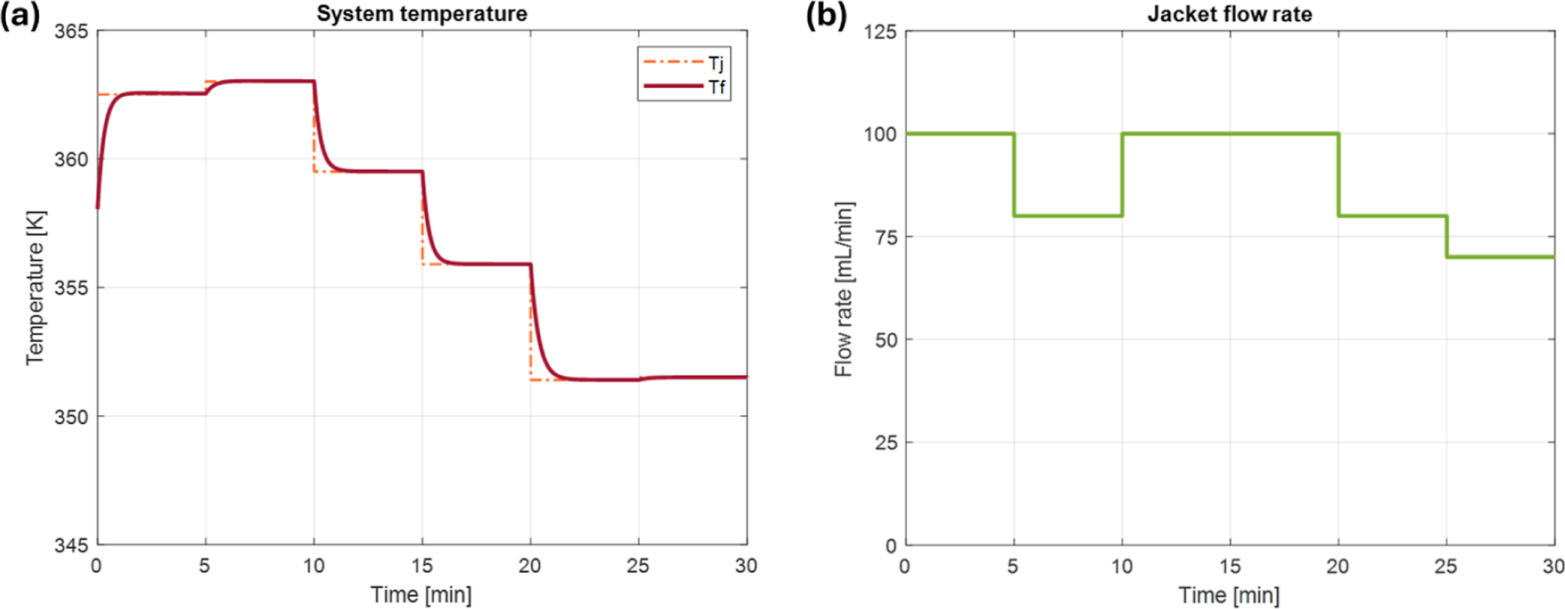
(a) The jacket temperature profile and the control result of the liquor-phase temperature, and (b) the feed flow rate profile.

**Figure 6:**
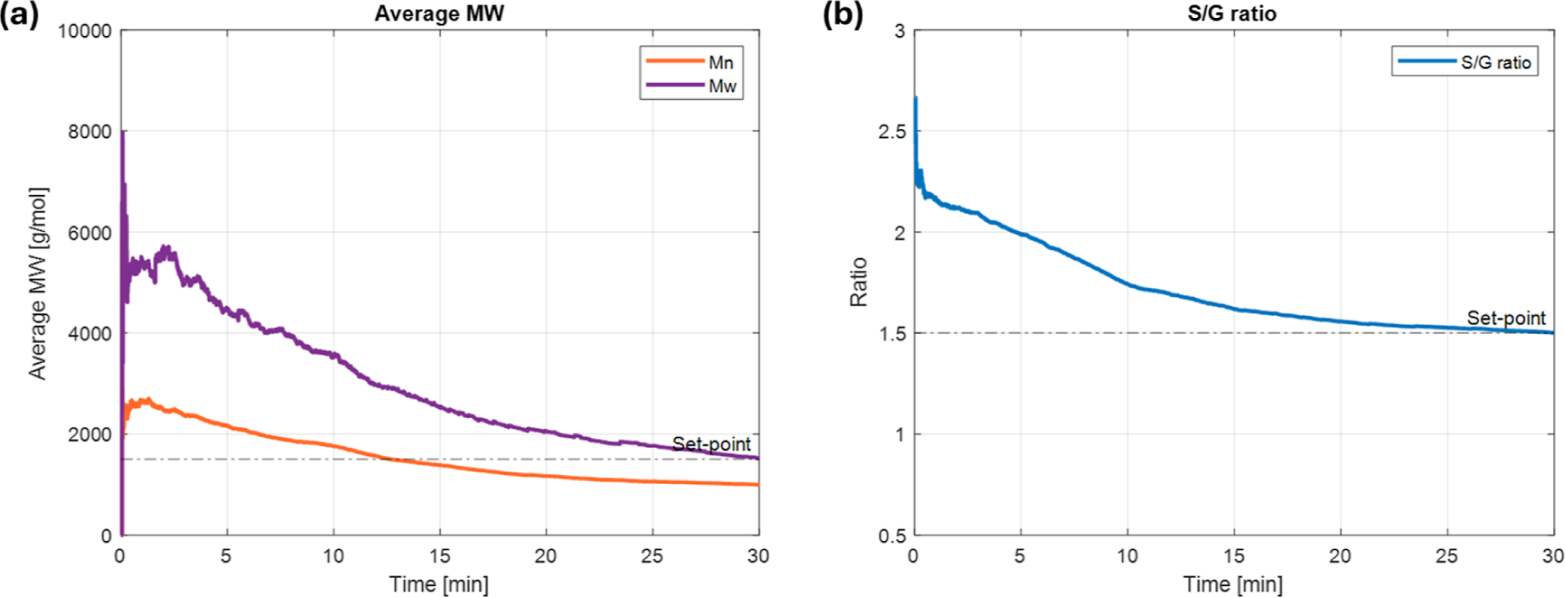
(a) The average molar mass, and (b) the S/G ratio converging to their respective set-points.

Subsequently, the system is cooled down to avoid an excessive drop in the S/G ratio against its set-point. According to the constraint (eq 12), which prevents drastic temperature change, *T*_f_ is decreased gradually. As a result, the S/G ratio is effectively converged to its set-point. The last segment shows a slight increase in the temperature, which can be attributed to fine-tuning for the final *M*_w_ control to its set-point, along with the S/G ratio. At the end of the reaction, the outputs are $\left[M_{w},S/G\right]=\left[1522,1.498\right]$ , which correspond to the control error of under 1.5% for both. These results showcase outstanding performance of the MPC.

This work demonstrates that the proposed accelerated kMC framework can offer sufficient computational speed to enable real-time MPC for lignin fractionation, while also achieving excellent closed-loop control performance under the considered conditions. The success of this approach highlights its potential for integration in advanced biorefinery operations that require rapid and robust control of lignin properties.

While the above results establish a foundation for practical deployment of the control strategy, several important directions remain for future research. Particularly, investigations that consider the influence of factors from the actual experiments, including process noise, sensor delays, and the detailed heat transfer characteristics of the reactor, will be essential to assess the true robustness and industrial applicability of the control scheme. Further, systematic robustness testing under various disturbances and operational uncertainties would provide deeper insights into the practical stability and reliability of the controller in real, scale-up processes.

## 4 Conclusion

This study presented a threshold-filtered kMC framework that significantly accelerates the multiscale simulation of lignin fractionation without compromising stochastic fidelity. By incorporating an Arrhenius-based activation energy threshold and event-specific data structures, the proposed algorithm eliminated kinetically irrelevant events and redundant rate evaluations, achieving an order-of-magnitude reduction in computational cost compared to the conventional kMC simulations. The developed model successfully captured key molecular-scale dynamics, including depolymerization, condensation, and demethoxylation, under varying temperatures, accurately predicting the evolution of MWd and S/G ratios with minimal deviation from high-fidelity references.

The enhanced computational efficiency enabled seamless integration with the MPC framework, demonstrating real-time regulation of lignin molecular characteristics through temperature and feed-flow manipulation. This coupling establishes a foundation for process-intensified lignin valorization, where dynamic optimization can be performed at both molecular and reactor scales. Furthermore, the purely algorithmic acceleration strategy removes dependence on pretrained data sets, providing generalizability across reaction conditions and lignin sources. Overall, the methodology presented herein offers a scalable, physics-based modeling platform that bridges detailed stochastic simulation with real-time process control, thereby advancing the optimization of lignocellulosic biorefineries.

## Supplementary Material



## Notes

Overall simulation workflow; activation energy calculations; comparison on computational efficiencies and predictive accuracies (PDF)
