# Supplementary material for: Threshold-Filtered Kinetic Monte Carlo Simulation for Real-Time Simulation and Control of Biomass Fractionation
Source: Ind Eng Chem Res. 2026 Mar 13;65(11):6092–102. doi: 10.1021/acs.iecr.5c04698 (PMC13022807; doi:10.1021/acs.iecr.5c04698)
Supplement: Supplementary file 1 [file ie5c04698_si_001.pdf]

Supporting Information

Threshold-Filtered Kinetic Monte Carlo Simulation for Real-Time Simulation and Control of Biomass Fractionation

Juhyeon Kim[†,‡,¶], Jiae Ryu[§], Qiang Yang[‖], Chang Geun Yoo[§], and Joseph Sang-Il Kwon[*,†,‡,¶]

[†] *Artie McFerrin Department of Chemical Engineering, Texas A&M University, College Station, TX 77845 USA*

[‡] *Texas A&M Energy Institute, Texas A&M University, College Station, TX 77845 USA*

[¶] *William G. Lowrie Department of Chemical and Biomolecular Engineering, Columbus, OH 43210 USA*

[§] *Department of Chemical Engineering, State University of New York College of Environmental Science and Forestry, Syracuse, NY 13210, USA*

[‖] *School of Packaging, Michigan State University, East Lansing, MI 48824, USA*

[*] E-mail: kwon.677@osu.edu

S1. Overall simulation workflow

The schematic illustration for the proposed simulation algorithm is shown in Fig. S1. At the beginning of the simulation, the system is initialized by generating lignin chains with randomly assigned S/G sequences until the total mass of the generated chains reaches the prescribed lignin content. For each chain, the molar mass and S fraction are calculated directly from its S/G sequence. In addition, the activation energies for depolymerization, E_{dep}, are evaluated for all inter-monolignol bonds using a database derived from density functional theory (DFT) calculations. The detailed procedure for E_{dep} assignment is described in detail in our previous publication [1].

To restrict depolymerization rate evaluations to kinetically relevant events, cleavable sites are identified using an activation energy threshold, ΔE_{th}. For a given chain i, the algorithm first determines the scission site with the lowest activation energy, $E_{dep,i,low}$. All other sites whose activation energies exceed $E_{dep,i,low} + \Delta E_{th}$ are then classified as uncleavable and excluded from the candidate event list. Because condensation reactions depend only on chain length, this screening procedure is applied exclusively to depolymerization events.

Upon generation of each chain, the following properties are stored in a structure for subsequent use: S/G sequence, chain length, molar mass, E_{dep}, depolymerization candidate, and S fraction (f_S). A simple illustration is shown in Fig. S2.

The chip phase and the liquor phase exchange lignin chain entries through delignification and redeposition processes. Within the liquor phase, dissolved lignin chains undergo microscopic reactions, including depolymerization, condensation, and demethoxylation, and the resulting changes are updated.

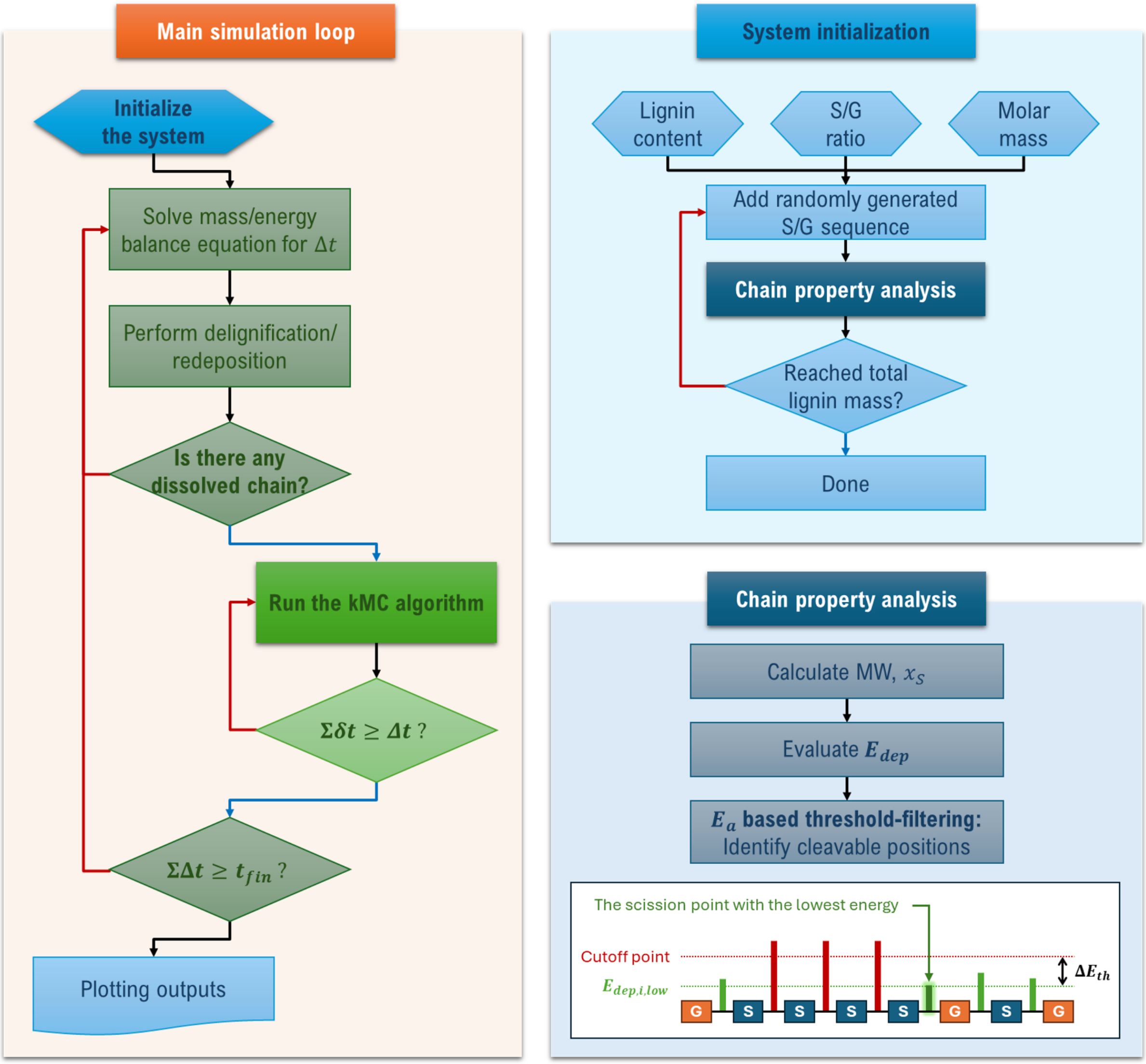

Fig. S1: A schematic illustration of the overall simulation process.

As illustrated in Fig. S2, condensation reactions depend on chain length; therefore, candidate reactions are identified by querying the corresponding chain-length entries. Depolymerization reactions are restricted to pre-identified cleavable sites, for which reaction rates are evaluated and events are executed accordingly. In addition, demethoxylation rates are computed using the S fraction values stored in advance for each chain. The schematic illustration of the kMC algorithm is shown in Fig. S3.

S2. E_a changes under the different chain lengths

The detailed procedure for calculating activation energies for each microscopic event is explained in our previous article [1] in detail. Note that E_{dep} values are used as a discrete value for each S/G configuration. The detailed overview of E_{con}, which is represented as a function of the

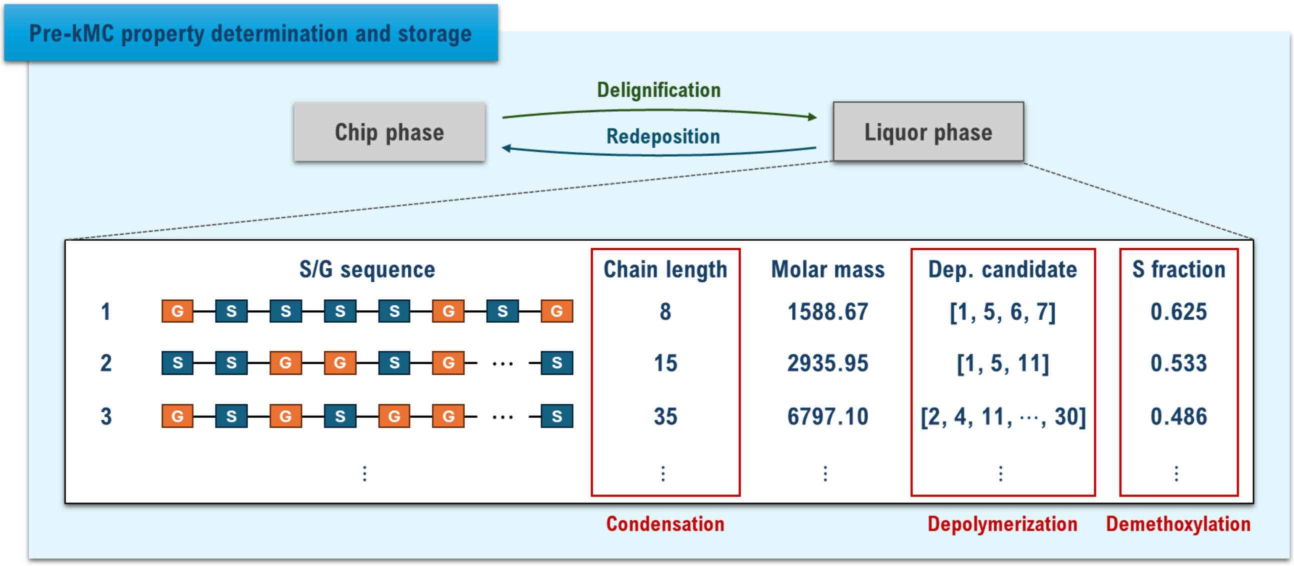

Fig. S2: An illustration of the integrated data structure.

temperature and the molar mass of a condensed chain, is shown in Fig. S4.

S3. Detailed comparison on computational efficiency

Detailed comparison of the computational overheads associated with the high-fidelity kMC (HF-kMC) [1], ANN-accelerated kMC (AA-M-kMC) [2], and the proposed threshold-filtered kMC (TF-kMC) is shown in Fig. S5. In the HF-kMC framework, all possible microscopic events are explicitly evaluated throughout the entire simulation, including kinetically negligible pathways. As a result, the computational demand increases drastically as the lignin population grows, leading to an overwhelmingly large CPU time dominated by microscopic event evaluation.

The AA-M-kMC framework alleviates this bottleneck by parametrizing heterogeneous lignin populations using Gaussian mixture models (GMMs) and estimating microscopic rate distributions through pre-trained artificial neural networks (ANNs). By decomposing the microscopic layer into GMM parametrization, rate distribution inference, and even execution, the overall computational cost is substantially reduced compared to HF-kMC.

In contrast, the TF-kMC framework achieves algorithmic acceleration by directly reducing the number of microscopic rate evaluations. Analysis of the microscopic rate distribution reveals that conventional kMC algorithms unnecessarily evaluate a large number of kinetically irrelevant reactions, causing the computational complexity to scale superlinearly with the number of chains. The threshold-filtering algorithm eliminates such events based on an Arrhenius-type activation energy criterion, thereby significantly reducing the computational burden. While the microscopic event execution time in TF-kMC is slightly higher than that of AA-M-kMC due to explicit rate evaluation, the total computational cost of the microscopic layer is markedly lower, as no

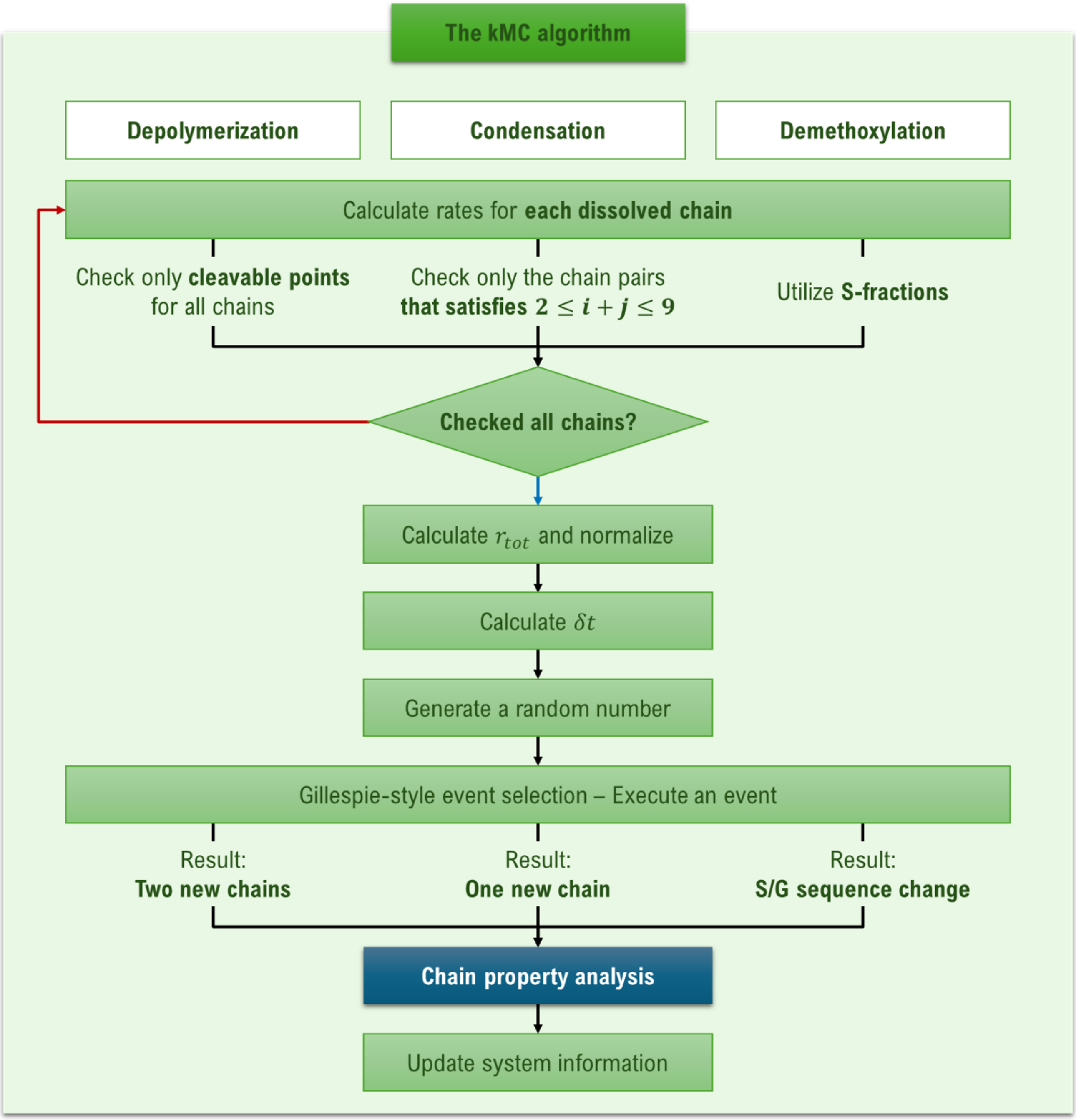

Fig. S3: A schematic illustration of the kMC simulation loop.

surrogate fitting or rate inference steps are required.

Apart from the microscopic layer, a modest increase in the chip configuration cost is observed for TF-kMC, which originates from the initial construction of structured dictionaries that store chain-specific properties. Nevertheless, by enabling direct access to pre-stored chain information rather than recalculating properties on the fly, the proposed algorithm achieves a substantial reduction in the overall simulation time while preserving full stochastic fidelity.

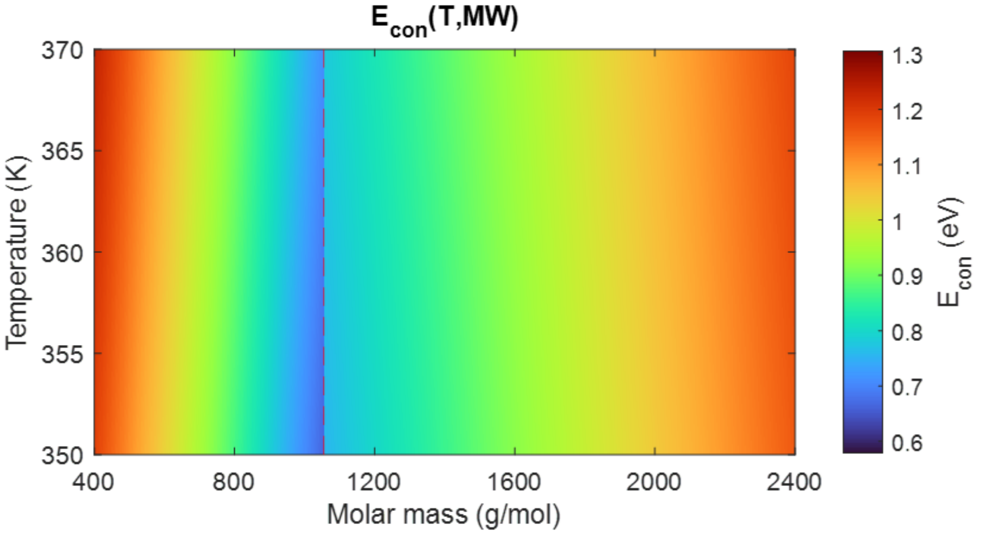

Fig. S4: The activation energy heatmap for condensation as a function of the temperature and the molar mass of a condensed chain.

S4. The prediction accuracy of the accelerated model

In Fig. S6, the simulation results from both high-fidelity kMC and threshold-filter kMC are plotted. While we ruled out the kinetically irrelevant events from the full event candidate, both model shows close match in terms of both the average MWs and S/G ratio, indicating excellent predictive capability of the accelerated kMC.

S5. Nomenclatures

A_{con}	Pre-exponential factor of condensation
A_D	Pre-exponential factor for delignification
A_{dem}	Pre-exponential factor for demethoxylation
A_{dep}	Pre-exponential factor of depolymerization
A_R	Pre-exponential factor for redeposition
$C_L(N_i)$	The concentration of the dissolved chain i
C_{P_c}	Heat capacity of the chip phase
C_{P_f}	Heat capacity of the liquor phase
$C_{P_{ext}}$	Heat capacity of the external heat source
ΔE_{th}	Threshold value for activation energy-based filtering
ΔH_R	Heat of the reaction
Δt	Macroscopic time step
δt	Microscopic time step
$E_{con,ij}$	Condensation activation energy for the dissolved chains i and j

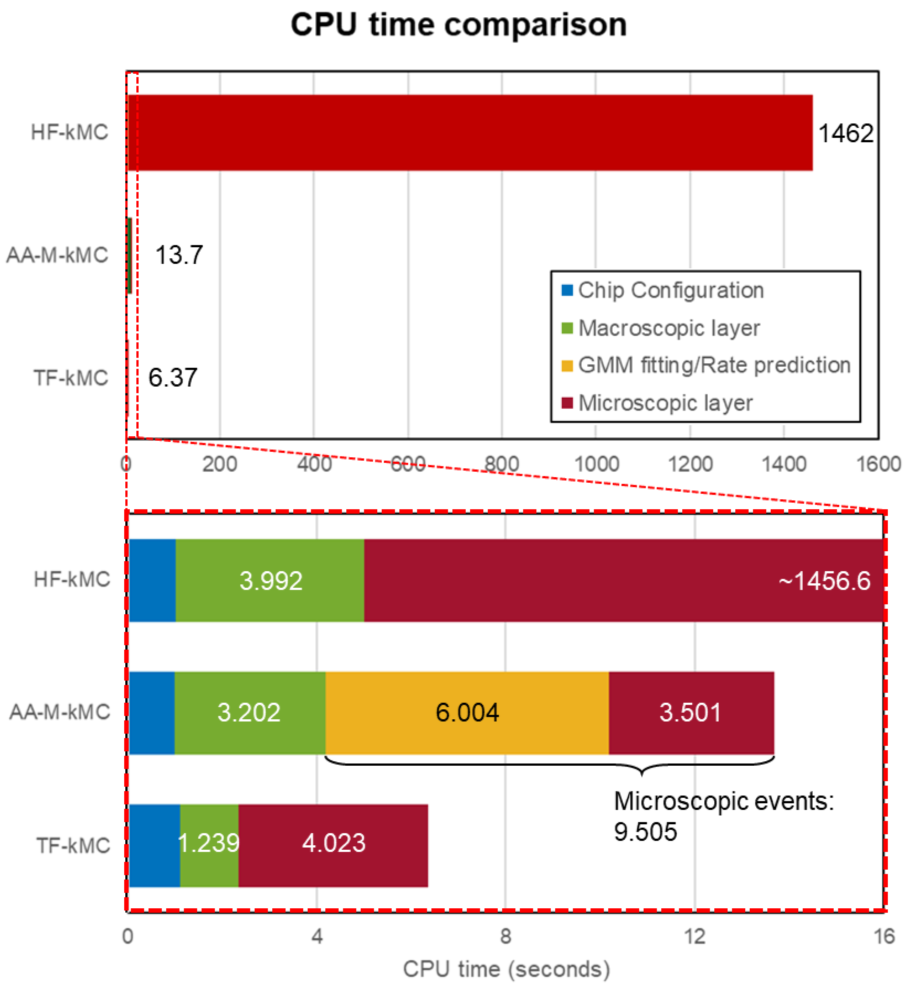

Fig. S5: Detailed comparison on computational overheads for HF-kMC, AA-M-kMC, and TF-kMC.

$E_{con,ij,low}$	The lowest condensation activation energy calculated from the DFT database
E_D	Activation energy for delignification
E_{dem}	Activation energy for demethoxylation
$E_{dep,i,low}$	The lowest depolymerization activation energy identified in a dissolved chain i
$E_{dep,im}$	Depolymerization activation energy for the m-th β-O-4 bond in a dissolved chain i
E_R	Activation energy for redeposition
f_{S_i}	The S-unit fraction in the chain i
$k_{con,ij}$	Condensation rate coefficient for the dissolved chains i and j
k_D	Delignification rate coefficient
$k_{dem,i}$	Demethoxylation rate coefficient for the dissolved chain i
$k_{dep,im}$	Depolymerization rate coefficient for the m-th β-O-4 bond in a dissolved chain i
k_{fast}	Rate coefficient for the faster reaction

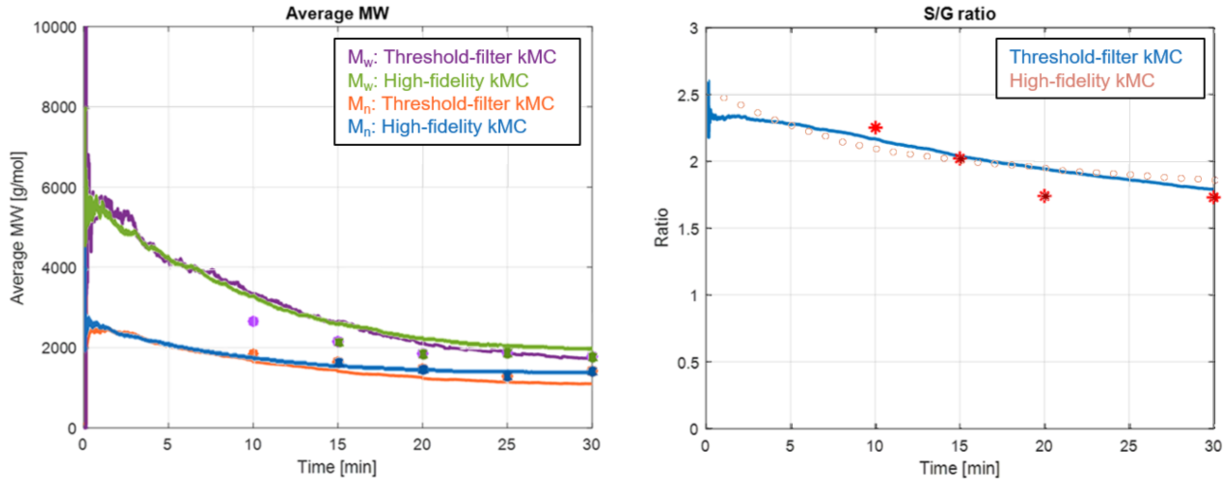

Fig. S6: The outputs from both HF-kMC and TF-kMC for average MW and S/G ratio at 353 K.

k_R	Redeposition rate coefficient
k_{slow}	Rate coefficient for the slower reaction
L_c	The mass of lignin in the chip phase
L_d	The mass of dissolved lignin in the liquor phase
M_c	Total mass of the chip phase
$\dot{M}_{ext}$	Mass flow rate of the external heat source
M_f	Total mass of the liquor phase
MW_{i+j}	The combined molar mass of the dissolved chains i and j
M_n	The number-average molar mass
M_w	The weight-average molar mass
N	The number of the prediction horizon in the MPC framework
R	Universal gas constant
$r_{con,ij}$	Condensation rate for the dissolved chains i and j
r_D	Delignification rate
$r_{dem,i}$	Demethoxylation rate for the dissolved chain i
$r_{dep,im}$	Depolymerization rate for the m-th β-O-4 bond in a dissolved chain i
r_R	Redeposition rate
r_{tot}	The sum of all microscopic rates at a given moment
T_c	Temperature of the chip phase
T_{ext}	Temperature of the external heat source
T_f	Temperature of the liquor phase
U	The overall heat transfer coefficient
$\mathbf{X}_p$	The controlled outputs in the MPC framework
ξ	A uniformly distributed random number for kMC execution

ω_p	A weighting constant for control output p

Literature Cited

[1] Lee, C. H.; Kim, J.; Ryu, J.; Won, W.; Yoo, C. G.; Kwon, J. S.-I. Lignin structure dynamics: Advanced real-time molecular sensing strategies. *Chemical Engineering Journal* **2024**, *487*, 150680.

[2] Kim, J.; Ryu, J.; Yang, Q.; Yoo, C. G.; Kwon, J. S.-I. Real-Time Model Predictive Control of Lignin Properties Using an Accelerated kMC Framework with Artificial Neural Networks. *Industrial & Engineering Chemistry Research* **2024**, *63*, 20978–20988.
